# Addressing Malnutrition: The Importance of Political Economy Analysis of Power

**DOI:** 10.34172/ijhpm.2020.250

**Published:** 2020-12-16

**Authors:** Helen Walls, Nick Nisbett, Amos Laar, Scott Drimie, Shehla Zaidi, Jody Harris

**Affiliations:** ^1^Department of Global Health and Development, Faculty of Public Health and Policy, London School of Hygiene and Tropical Medicine, London, UK.; ^2^Health and Nutrition Cluster, Institute of Development Studies, University of Sussex, Brighton, UK.; ^3^Department of Population, Family and Reproductive Health, University of Ghana, Accra, Ghana.; ^4^Division of Human Nutrition, Department of Global Health, Faculty of Health and Medicine Sciences, Stellenbosch University, Stellenbosch, South Africa.; ^5^Department of Community Health Sciences, Aga Khan University, Karachi, Pakistan.

**Keywords:** Power, Nutrition, Malnutrition, Double Burden of Malnutrition, Food, Food Systems

## Abstract

**Background:** The exercise of power is central to understanding global health and its policy and governance processes, including how food systems operate and shape population nutrition. However, the issue of power in food systems has been little explored empirically or theoretically to date. In this article, we review previous work on understanding power in addressing malnutrition as part of food systems that could be used in taking this issue further in future food systems research. In particular, we examine why acknowledging power is vital in addressing food systems for better nutritional outcomes, approaches to assessing power in empirical research, and ways of addressing issues of power as they relate to food systems.

**Methods:** We undertook a narrative review and synthesis. This involved identifying relevant articles from searches of PubMed and Scopus, and examining the reference lists of included studies. We considered for inclusion literature written in English and related to countries of all income levels. Data from included articles were summarized under several themes.

**Results:** We highlight the importance of *acknowledging* power as a critical issue in food systems, present approaches that can be taken by food-systems researchers and practitioners in *assessing* power to understand the ways in which power works in food systems and wider society, and present material relating to *addressing* power and developing strategies to improve food systems for better nutrition, health and well-being.

**Conclusion:** A range of research approaches exist that can inform examination of power in food systems, and support the development of strategies to improve food systems for better nutrition, health and well-being. However, there is considerable scope for further work in this under-researched area. We hope that this review will support the necessary research to understand further power in food systems and drive the much-needed transformative change.

## Background

The exercise of power is central to understanding global health and its policy and governance processes, including how food systems operate and shape population nutrition.^1-3^ However, and despite the substantial burden of malnutrition in countries globally, power in food systems is an issue that has been little explored empirically or theoretically to date.^4^ Thus in this article we review previous work on understanding power in addressing malnutrition as part of food systems that could be used in taking this issue further in future food systems research. In particular, we examine the reasons why acknowledging power is vital in addressing food systems for better nutritional outcomes, approaches to assessing power in empirical research, and ways of addressing issues of power as they relate to food systems.

Malnutrition in all its forms is the leading contributor to the global burden of disease, responsible for more deaths and days lost to ill-health than any other single factor.^5^ The various forms of malnutrition contributing to this alarming situation are interrelated: at one end of the spectrum are issues of undernutrition such as stunted growth and thinness; at the ‘other end’ are obesity and its attendant non-communicable diseases; and throughout are issues of micronutrient deficiencies. International development efforts have historically focused on undernutrition and micronutrient deficiency. Obesity and other non-communicable diseases have generally been addressed separately. Over the last decade, however, public health experts have increasingly considered malnutrition in all its forms as a range of disorders that share common causes in poor diets and lack of healthcare. Malnutrition is now usefully described as a double (or even triple) burden, with undernutrition and deficiencies coexisting with obesity and other non-communicable disease in the same countries, households and individuals.^6,7^ The latest estimates suggest that a third of countries have significant levels of undernutrition as well as obesity and non-communicable disease,^8^ thus a ‘double burden of malnutrition.’

At the end of 2019 the *Lancet* journal released another of its influential nutrition series, this one on the double burden of malnutrition – the Lancet Series on the Double Burden of Malnutrition. This new and timely series presents a broad range of evidence emphasising the interconnected nature of different forms of malnutrition and the biological, economic and policy drivers and implications.^9^ However, we suggest that to address the full spectrum of malnutrition requires a focus beyond malnutrition’s immediate causes – diet and disease – and even beyond their underlying determinants – food systems – to understand characteristics of *power* as a fundamental driver of malnutrition globally. Thus, inspired by our observations at the Series launch of the ways in which power was included and not included in research and debates of these issues, we undertook (January to May 2020) a narrative review and synthesis of existing literature relating to power in addressing issues of malnutrition in food systems, and also of literature elaborating empirical approaches for understanding power, that could be used for expanding and advancing power analyses in future food systems research. The narrative review and synthesis methodological approach is considered particularly useful for obtaining a broad perspective or overview of a topic, without attempting to identify all relevant literature.^10^

As part of this narrative review and synthesis, we searched PubMed and Scopus for articles using combinations of the keywords ‘power,’ ‘nutrition,’ ‘malnutrition,’ ‘double burden of malnutrition,’ ‘food,’ ‘food systems,’ ‘health’ and ‘health systems.’ We evaluated all types of literature including reviews, research articles, case-studies, and commentaries, and considered for inclusion literature related to countries of all income levels. We also examined the references of included studies to identify additional sources. Only studies written in English were included. Data from the included articles are summarized under the following headings: acknowledging power in food systems; assessing power in food systems; and addressing power in food systems. We conceptualise food systems broadly, using the definition of the Food and Agriculture Organization: ‘food systems encompass the entire range of actors and their interlinked value-adding activities involved in the production, aggregation, processing, distribution, consumption and disposal of food products that originate from agriculture, forestry or fisheries, and parts of the broader economic, societal and natural environments in which they are embedded.’^11^ All study authors contributed to the review and synthesis process, including interpretation of findings, and reviewed iterations of the final manuscript.

## Results

###  Acknowledging Power in Food Systems

Power is exercised throughout global health, and is central to understanding how food systems operate, including the governance of nutrition through policy and regulation.^1-3^ Food systems comprise vast networks of actors – including governments, businesses, people, and coalitions – connected in a variety of intricate ways, all with different aims and approaches to realising their own interests, whether that be simply survival, well-being or the enjoyment of cooking and eating; pursuing a food-related livelihood; providing for members of a community, or, more broadly, citizens; or capturing resources (profits, subsidies, land etc). We use the word *power* to describe what allows or prevents these actors from realising these interests; why and how systems including food systems tend to favour particular groups over others over time; why resources accrue to some and not others; who gets to set societal norms and beliefs and how this affects individuals or groups; and how people may struggle against and resist this.^1-3^ The issue of power within food systems has been little explored empirically or theoretically to date, with researchers and practitioners who focus on various forms of malnutrition only for the most part starting to examine this topic.^4,12^

The London launch of the Lancet Series on the Double Burden of Malnutrition^9^ in December 2019 provided a fascinating insight into how power asymmetries in global health are increasingly acknowledged in expert discussions, but are not yet front and centre of actual research. It was notable that in the discussion of the launch, the assembled experts moved away from discussing the data and evidence relating to the double burden of malnutrition to discussing its underlying cause: the issue of power. As a global nutrition community, we will not be able to sustainably address malnutrition in all its forms without focussing on its basic causes – including, critically, issues of power. Such issues *are* addressed by the Series (largely in the first paper by Popkin et al^13^), and also receiving mention in some of the subsequent papers), but not nearly as centrally as they appeared in the ensuing debate. Addressing power requires moving from its debate amongst public health academics and practitioners to power being an issue at the core of public health research and practice.

Discussions at the Series launch centred on a particular form of power – the power of large companies who market unhealthy foods and beverages, or lobby in backrooms against public health measures. Whilst a core interest of the global health community is to address malnutrition resulting from an inadequate food system, others in the food system have different interests. Critically, corporate entities are heavily incentivised by profit maximisation and related efforts to reduce (public health) regulation. We agree with the importance of understanding influence of large private actors^14,15^ but we also highlight here that power imbalances permeate all relationships concerning nutrition. This is in regard to malnutrition in all its forms, and includes the influence of prominent philanthropists and other development actors wielding financial, epistemic and normative power^ [1],2,16^ the daily inequities faced by and within the world’s poorest households ^17,18^, and the lack of representation of the poor and marginalised in systems of local and national government and health governance.^19^

###  Assessing Power in Food Systems

Understanding differential power and influence in food systems including in food and nutrition governance requires political economy research approaches. These concern themselves with how power dynamics emerge through competing stakeholder priorities, and perpetuate systems – including food systems, as well as wider social and economic systems – that favour the powerful and let them set the rules of the game; or ossify systems so that things stay the same even though individuals may move on or even be replaced by others with competing interests.^20-23^

Popular conceptualisations of power are as a simple force applied authoritatively or coercively, but wider political research stresses that power dynamics operate diffusely and with complexity within the relationships, norms and narratives that structure policy or programmatic decision-making.^20,24-27^ Whilst there are numerous summaries of theories of power, each with its disciplinary approach, we set out here some common conceptualisations with application to examining food systems.

As with lay conceptions, power was conceptualised originally within political science as a negative and limiting force of ‘power over;’ as the ability of some groups to gain control over resources or political systems; their ability to control key institutions, set the agenda and exclude others’ concerns from that agenda. But power has also been theorised as a more positive and enabling force as ‘power to,’ including power ‘to resist,’ or ‘power with,’ a collective power, ‘power within,’ relating to self-efficacy, or ‘power for,’ emphasising collective vision.^28^ In addition, some traditional definitions of power view it as held by distinct actors in key economic or political institutions broadly defined (elitism), or distributed between different groups (pluralism), according to how they access resources and enter coalitions. The ‘structuration’ perspective shifts the focus of power from something held by actors, to something that operates at a system level,^29,30^ and emphasises the ‘predetermination of the behavioural options of political decision-makers’ by the structure of the system.^30^ Such different forms of power are not mutually exclusive; they can exist all at once, are interrelated, and their dynamics change over time, shaped themselves and perhaps also shaping the systems and structures in which they exist.

Importantly, there are not only different forms power, but power itself operates in different spaces and at different levels. Of particular relevance here is Steven Lukes’ work on the three ‘dimensions’ of power,^31^ extended into the Power Cube model by John Gaventa.^32,33^ People and institutions exert their power openly but also behind ‘closed’ doors, or in privileged ‘invited’ spaces (such as within global food and nutrition fora or trade negotiations^34^) – challenged only when these spaces of power are contested and perhaps ‘claimed,’ for example through protest and public displays of outrage, including food riots.^35^ Furthermore, power can be exercised in different forms, including the aforementioned and ‘hidden’ ways in which agendas are set in non-inclusive ways and through its ‘invisible’ role in shaping our beliefs and ideologies, and thus, conceptions of what types of political decisions are legitimate.^31^ These different levels of power (global, national, local); spaces of power (closed, invited, claimed); and forms of power (visible, hidden, invisible) can all be assessed.^32,33^ An example of an additional tool for such assessment is the framework by Milsom et al developed for analysing power in public health policy decision-making as well as for *non*-decisions, in an article examining how corporate power and international trade shape policy-making including in relation to ultra-processed foods.^24^ Political economy analyses of power go beyond simply listing the different actors involved in the policy process. Extending upon the work of Lukes, Gaventa and others, the framework of Milsom et al differentiates between forms, mechanisms, dimensions and outcomes of power in public health policy-making.^24^ Assessments of power, informed by such frameworks, can help to identify who has the power to effect change (or maintain the status quo), to what purposes and in whose interests, as well as the forms of power exercised, its mechanisms, its dimensions (eg, levels and spaces), and its outcomes. Notably, for change to happen in ways that do not simply perpetuate forms of hidden or invisible power, in closed and narrowly invited spaces, activism and advocacy might need to focus on multiple forms of power, in multiple spaces and at a variety of spatial scales, to be effective.^28,32^

In disciplines such as political science or anthropology where power is a central concept, detailed studies including comparative, historical, longitudinal and ethnographic methods have helped shed light on the dynamics of power, including the complexity of the beliefs and interests that lie behind major policy decisions.^36,37^ Recognising that this kind of in-depth research is not always possible, but that comprehensive assessments of power are fundamental to informing the creation of more accountable policy processes, complementary frameworks and approaches have been developed. These include participatory power mapping, following from the Power Cube model,^32,33^ which has been used by non-governmental organisations, civil society and activist organisations around the world to structure power analysis. Questions to be probed by this method^38^ include:

- *WHO? Individual actors, organisations, and institutions involved in policy processes* (both formally and informally): Whose voices are the loudest and who struggles to be heard? Who is directly or indirectly helping these voices to be heard? What interests do these actors represent? Via what professional, political, educational, social, kinship and/or ethnic networks are they connected?
- *WHERE? Levels, spaces and context in which decisions are made:* At what levels and in which spaces are decisions relevant to policy or implementation being made and opinions articulated (household, community, national, global)? What is the wider context for these? Are policy spaces open, closed, invited (open only to certain groups), contested, or claimed (ie, by protest or by setting in place alternative policy solutions)? Are formal policy spaces more dominant, or perhaps more informal settings?
- *WHAT? Sectors, issues, and forms of power:* What is the construct of nutrition by lead actors and hence the change being effected? What are the underlying motivations and interests driving delivery? Which aspects of marginalisation or equity are being addressed, if at all? In what ways are actors operationalising their power? Are actors operating in ways that are visible; hidden (with items kept off the agenda); or invisible (embedded in belief and knowledge systems)? What are the assumed norms and narratives around current nutrition policy areas and how do they contribute to hidden and invisible forms of power?
- *HOW? Strategies, platforms and models for creating change:* What strategic approaches are used for responding to the above? What governance structures are in place for power sharing amongst multiple stakeholders? What is the logic behind the choice of partners, allies and actors? What is the role and strategy of different actors in the work they support or carry out? What incentives and disincentives are shaping stakeholder cooperation and power sharing? What type of leadership is being undertaken, for example: sole, distributive?

Power dynamics (and imbalances) within global and national food systems have led to many ineffective and incoherent policy decisions,^39-41^ decisions which have failed to address malnourishment, leaving many people under-served, under-voiced, and unable to hold the powerful to account. With policy-making, although rational, linear models for understanding the policy process (eg, proceeding through the stages of agenda setting, policy formulation and legitimisation, implementation, evaluation and review), and the relationships between evidence and policy can be useful heuristics to support basic conceptualisation, policy processes rarely follow such a path. Rather, policy-making is a non-linear process shaped by political and other wider contextual factors, and power is a critical concept to understand policy processes and decisions.^42-48^ A major data and analytical gap for improving accountability in food and health systems and nutrition policy processes relates to the type of more comprehensive power analysis described here. Challenging the activities which lead to suboptimal outcomes for food systems, including for population nutrition, calls for rigorous analysis of how power operates; only then can suitable structures be designed to ensure a more transparent and responsible exercise of power in those fora mandated to make responsive and effective decisions in the interests of tackling malnutrition.^49-51^ Epidemiological and other evidence is essential for understanding how food systems are transitioning, but understanding how power operates within and beyond these systems is critical for understanding drivers and enacting change.

###  Addressing Power in Food Systems

Globally, public health and nutrition communities have long been aware of power imbalances when considering, for example, the history of the marketing of breastmilk formula,^52^ the undue influence of processed food manufacturers over policy to address obesity,^53^ or the power and influence in global trade agreements of corporate actors and of larger and wealthier countries and regions over those smaller and more resource-constrained.^54,55^ Academic work in this area has been critical to improving understanding of the power dynamics shaping food systems and nutrition outcomes, and helping to identify factors that support or restrict implementation of nutrition policy and programmes. However, considering analyses of power also suggests need for new approaches to addressing such food system problems – and the need for much further work in this area.

The analyses of power that we propose would assist with identifying, understanding, and providing the evidence base from which to address the challenges and opportunities at critical points of the current global food system. These analyses would include work on equity and marginalisation, policy processes at multiple scales and system levels including their interconnections, and macro-structural drivers such as trade and economic policy and the role of private sector entities. Previous work on understanding power to achieve nutritional goals indicates, for example, that multi-sectoral platforms and approaches require strong championing and coordination supported by joined-up funding and targets to counter disincentives to collaborative working, as seen in Pakistan^56^ and Peru.^57^ There is also indication that power issues in regard to decision-making and resources between central and local governments can compromise the delivery of food and health systems, as seen in Pakistan,^58^ whereas mutual recognition of institutional benefits such as electorate popularity can forge cooperation between local actors and vertical programs as seen in Brazil and India.^59^ Notwithstanding need for further research,^60^ studies from a range of countries suggest that civil society activism and other participatory approaches creating bottom-up demand for better nutrition and health have challenged power structures in various contexts.^21,60^ With this there are particularly lessons to learn from existing and successful food-based movements, such as Indian’s Right to Food Campaign,^61^ or the role of ‘peasant-led’ or worker-led struggles in Brazil’s wider and successful policy of ‘Zero Hunger.’^62^ Further work in this area would build on this, and likely lead to the development of new and more nuanced understandings and suggestions.

Analyses of power in national policy processes generally highlight the importance of the state in regulation, oversight, and ensuring human rights are protected in regard to population nutrition and providing the essential public services and basic infrastructure that have been associated with better health and nutrition outcomes.^63^ If commensurate with accountability mechanisms to support governance processes, constrain the influence of corporate and other actors with conflicts of interest in public policy development, and reinforce civil society engagement with achieving healthy food goals,^53^ this could form a social compact between the state and its citizens. In times of plenty, abundant and well-distributed food helps to maintain the social contract between citizens and states, and so status-quo power relations; but in times of crisis, food can be the catch-point to trigger reorganisation of power.^64,65^ There is also the challenge of how to address the actions of a state that does not act in the interests of its people. Analyses of national policy processes have also helped to understand the challenges to developing policy informed by evidence, given that evidence is but one part of the policy process which is also informed by broader political considerations.^66-68^ However, such analyses, and work to understand the different food regimes that have shaped intertwined food and economic systems over centuries,^69,70^ often provide a reminder that the locus of power does not always rest with national governments. Whilst analyses of national policy processes remain important, in a globalised world, analysis of global power systems is also critical given limits to effectiveness of action from a single government or national system of governance.^21,63^

The globalisation of the food system brings to the fore newer issues of concentration of power outside of national governments, largely with global corporations^71^ but also through multi-stakeholder partnerships creating powerful lobbying groups^[2]^.^72^ Discussion at the Series launch included calls for a global code on the marketing of ultra-processed foods, to limit corporate power in a similar way that a global code on the marketing of breastmilk substitutes has tried to do,^73^ given increasingly clear evidence on the role of such foods in causing obesity, including amongst children.^74^ Whilst we support this approach to understanding and limiting undue corporate power, the global health community also needs to examine and address more broadly issues of stakeholder power within food systems, the trade-offs that inevitably result from public health, profit, and livelihood considerations of different actors and their power, and the varied effects on nutritional outcomes. Box 1 illustrates the issue of power at play in policy processes relating to the implementation of a tax on sugar-sweetened beverages (SSBs), highlighting the scope for power analyses applied to issues of food systems.

**Box 1. Power in Policy Processes Addressing Consumption of Sugar Sweetened Beverages**

Addressing the consumption of SSBs is, given the strong links to obesity and non-communicable disease, and dental caries,^75-77^ increasingly considered necessary by the public health community.^78^ There is particular advocacy and support for the introduction of taxes on SSBs, which evidence suggests, with some debate,^79^ reduces SSB consumption.^78,80^ In 2019, SSB taxes had been implemented in 40 countries globally.^81^ However, there is growing evidence globally that industry are heavily using their power and resources to invest in activities to oppose SSB regulatory measures, including implementation of national taxes on SSBs.^78,82^

In Africa, for example, only South Africa has successfully introduced an SSB tax. As Du et al describe, this tax, which came into effect in April 2018, experienced multiple delays in its implementation since 2016 when it was originally proposed. The fervent resistance from food companies to the implementation of the tax resulted in industry’s use of a number of tactics to counter the arguments made by advocates of the tax. At the same time, concerted advocacy was undertaken by civil society stakeholders in support of the tax.^78^ Despite the compelling rationale for governments to embrace SSB taxes, Morocco was forced in 2018 to repeal its SSB tax before its implementation in early 2019, due to pressure from the agri-food industry.^83^ A year on, the Moroccan parliament has considered a more watered-down version of the tax, although there has been no further progress to date.^84^

The experience of South Africa and Morocco with implementation of SSB taxes highlights clear tensions between stakeholders and strong power dynamics. The actions taken by corporate actors such as those illustrated by these two examples are increasingly documented in public health literature, including in regard to malnutrition-related policy processes in Africa,^25,85-89^ raising questions about asymmetries of power and other power dynamics between particularly public and private interests.^24,90-92^ Additionally, some analyses of power have been conducted specifically in regard to the political economy of SSB taxes and resistance to them.^82,93,94^ However, there is considerable scope for further and in-depth power analyses including through drawing on the approaches and frameworks described in this article, and in a greater number of contexts.

Abbreviation: SSB, sugar-sweetened beverages.

One existing framework with clear potential to redress power imbalances within and among states is the global human rights system. In 2019, over a hundred academics and activists signed a letter to the *BMJ* calling for rights-based guidance on food systems and diets, and suggesting that “international human rights law, institutions and mechanisms provide important opportunities for norm setting, advocacy and accountability which are currently underutilised” in addressing malnutrition.^95^ There is academic work describing what this framework might look like for nutrition,^96^ and how it might be considered of use to citizens and policy-makers.^97^ Successive United Nations Rapporteurs on food and nutrition have also called for placing power as a central consideration of food systems using human rights as a mechanism,^98^ which thus provides a focus not only on better outcomes but also on more equitable processes in the drive for better population nutrition.

Critically, such proposals for addressing malnutrition would be interventions in a complex system, and whilst changes to a system’s power distribution is effective for enacting systems change, work on complex systems shows that the most effective actions for achieving change involve addressing system goals and paradigm.^99^ This means understanding and addressing the goals, power structure, rules and culture out of which the system arises. The paradigm in which food and health systems exists is one of neoliberalism^24^ – an economic and political ideology with an inherent focus on reshaping the regulatory environment in favour of the market.^100,101^ The neoliberal perspective considers the responsibility for health behaviours and outcomes, including of relevance here food choice and nutritional outcomes, to lie with the individual, neglecting to consider the broader structural factors that shape these behaviours and outcomes.^102-104^ Developing the new food system that is so urgently needed to address malnutrition – one conducive to human, animal and environmental health, and responsive to the needs of people in high-, middle-, and low-income countries – will require new economic or public health models,^105^ and changes to stakeholder power and interests. Alternative system paradigms that may prove useful for achieving this include a human-rights framing, which has considerable traction globally,^95^ or framings on equity and inequality.^19^ However, the transformative change needed will involve a transdisciplinary approach appealing to – and capturing the imaginations of – a wide range of stakeholders.^105^ In the meantime, a focus on power is critical for understanding the influence of different actors within global food and health systems, and supporting development of effective strategies to address malnutrition – perhaps, ultimately, given the potential for such a focus to raise important questions about the very paradigm in which global food and health systems exist and support related advocacy, through something quite transformative.

## Conclusion

 This review highlights power as a critical issue in food systems, and describes approaches that can be taken by food system researchers and practitioners to assess power and, ultimately, use this understanding of power to address power imbalances and develop strategies to improve food systems for better nutrition, health and well-being. However, there is considerable scope for further work in this largely under-researched area. We hope that this review will assist food systems researchers and practitioners with acknowledging the importance of power in food systems, understanding some of the key power dynamics and their impact, and undertaking the necessary research to develop further understandings of power in food systems and drive the much-needed transformative change.

## Ethical issues

 Not applicable.

## Competing interests

 Authors declare that they have no competing interests.

## Authors’ contributions

 HW, NN and JH conceived of the article. HW led the writing of the manuscript. All study authors contributed to the review and synthesis process, including interpretation of findings, and reviewed iterations of the final manuscript.

## Authors’ affiliations

^1^Department of Global Health and Development, Faculty of Public Health and Policy, London School of Hygiene and Tropical Medicine, London, UK. ^2^Health and Nutrition Cluster, Institute of Development Studies, University of Sussex, Brighton, UK. ^3^Department of Population, Family and Reproductive Health, University of Ghana, Accra, Ghana. ^4^Division of Human Nutrition, Department of Global Health, Faculty of Health and Medicine Sciences, Stellenbosch University, Stellenbosch, South Africa. ^5^Department of Community Health Sciences, Aga Khan University, Karachi, Pakistan.

## Endnotes


^[1]^ ie, power not only over financial resources, but power to set research and policy agendas, or broader norms and ideologies.

^[2]^ We infer these as coalitions of power with likely negative impacts on food system outcomes for the poor and disempowered; but also note debates in the power literature about the possibility for resistance and collective action; as suggested by the earlier examples of Brazil and India. While these are national examples and while the excitement in earlier literature about the potential of more global-identity-based ‘new social movements’ has largely receeeded, we note from current writing new, youth-based movements appearing able to resist the status quo, such as those with increasing influence over the climate debate. Similar transnational examples within nutrition might include the earlier ‘baby killer’ campaigns against unethical breastmilk substitute formula marketing, which some (still challenged) success in taking on such forms of transnational corporate power; or the broader food sovereignty movement.

